# Network Analysis of the Relationships Between Symptoms, Functioning, and Sleep/Fatigue in Colorectal Cancer Patients

**DOI:** 10.1192/j.eurpsy.2024.1339

**Published:** 2024-08-27

**Authors:** J. Sim, J. Yu, Y. Yun

**Affiliations:** ^1^Department of AI convergence; ^2^Department of Psychiatry, College of Medicine, Hallym University, Chuncheon; ^3^Department of family medicine, Seoul National University, Seoul, Korea, Republic Of

## Abstract

**Introduction:**

Among the various patient experiences, cancer-related fatigue and sleep disturbances emerge as pivotal aspects that can substantially impact individuals’ quality of life. There exists a relative scarcity of research focusing on the intricate relationship between symptoms, functioning, fatigue, and sleep disturbances in colorectal cancer (CRC) patients.

**Objectives:**

In this context, the current research endeavors to apply advanced statistical methodologies to elucidate the complex relationships between symptoms, functioning, fatigue, and sleep disturbances. By exploring the intricate web of patient characteristics, clinical factors, psychosocial elements, this study aims to construct a holistic model that not only captures the nuances of colorectal cancer patients’ experiences but also uncovers potential avenues for intervention and support.

**Methods:**

In our cross sectional study, we administered the European Organization for Research and Treatment of Cancer Quality of Life Questionnaire Core 30 (EORTC QLQ-C30), the Quality of Life Questionnaire Colorectal Cancer Module (QLQ-CR29) to 987patients who were surgically-treated for CRC from the tertiary hospital from 2013 through 2018. To confirm the relationship between symptoms of CRC patients, univariable logistic regression was used to examine the potential relationship between independent variables and the occurrence of fatigue and sleep disorders. Least Absolute Shrinkage and Selection Operator (Lasso) was used for variable selection. The selected variables were then applied to a multivariate logistic regression analysis to examine the most influential predictors of fatigue and sleep disturbance. Finally, gaussian graphical models (GGM) were used to identify potential interactions between characteristics, symptoms, functioning, with fatigue, and sleep disturbances in CRC. In this study, Directed Acyclic Graph (DAG) was used to identify causal dependancy and path of variables.

**Results:**

About 10.4% of study participants reported experiencing fatigue. Sleep problems were reported by 15.8% of the study participants. Multivariable logistic regression analysis using Lasso showed that sleep problem (odds ratio [OR]=2.34; 95% CI, 1.03-5.31), physical, role, and emotional functioning, pain, dyspnoea, and appetite loss were significant predictors of fatigue, while emotional functioning, dyspnoea, and appetite loss were significant predictors of sleep problem. The variables that were directly linked to fatigue were role functioning, emotional functioning, dyspnoea, appetite loss, body image and trouble with taste. The variables that were directly linked to sleep problem were emotional functioning and appetite loss.

**Conclusions:**

In conclusion, there were complex relationships between symptoms, functioning, fatigue, and sleep disturbances. The symptom network of CRC patients showed different patterns toward fatigue and sleep.

**Disclosure of Interest:**

None Declared

